# Prevalence, severity and impacts of breathlessness in Indian adults: An exploratory, nationally representative, cross-sectional online survey

**DOI:** 10.1371/journal.pgph.0002655

**Published:** 2024-05-02

**Authors:** Slavica Kochovska, Rajam Iyer, Sungwon Chang, Diana Ferreira, Vanessa N. Brunelli, Irina Kinchin, Danny J. Eckert, Joseph Clark, Jacob Sandberg, Magnus Ekström, David Currow, Sujeet Rajan

**Affiliations:** 1 Faculty of Science, Medicine and Health, University of Wollongong, Wollongong, New South Wales, Australia; 2 P.D. Hinduja Hospital and Medical Research Centre, Mumbai, Maharashtra, India; 3 IMPACCT, Faculty of Health, University of Technology Sydney, New South Wales, Australia; 4 Centre for Health Policy and Management, Discipline of Public Health & Primary Care, School of Medicine, Trinity College Dublin, University of Dublin, Dublin, Ireland; 5 College of Medicine and Public Health, Flinders University, Adelaide, South Australia, Australia; 6 Wolfson Palliative Care Research Centre, Hull University, Hull, United Kingdom; 7 Department of Clinical Sciences Lund, Faculty of Medicine, Respiratory Medicine and Allergology, Lund University, Lund, Sweden; 8 Bombay Hospital and Medical Research Centre, Mumbai, Maharashtra, India; The George Institute for Global Health, UNSW, AUSTRALIA

## Abstract

There are no known estimates of the prevalence, severity and impacts from breathlessness in low- and middle-income countries. This study aimed to explore the prevalence, severity, self-attributed underlying conditions and impacts of *breathlessness limiting exertion* in community-dwelling adults in India. This exploratory, population-based online survey recruited a pre-planned sample of 3,000 adult respondents stratified by age, sex and rurality (quotas as per the 2011 Indian National Census). Measures included: demographics; *breathlessness limiting exertion* (modified Medical Research [mMRC] scale); health-related quality of life (EQ-5D-5L); and disability (World Health Organisation’s Disability Assessment Schedule 2.0 12-item questionnaire [WHODAS-12]). Respondents (n = 3,046) had a mean age of 38 years (SD 15); 57% were male, 59% lived in rural areas and 33% had completed 12^th^ grade. *Breathlessness limiting exertion* (mMRC ≥1) was reported by 44%, mostly attributed to poor nutrition (28%), lung conditions excluding tuberculosis (17%) or anaemia (13%). Compared to those without breathlessness, a higher proportion of people with breathlessness (mMRC ≥1) reported problems across all EQ-5D-5L dimensions. Most people reporting breathlessness (81%) indicated the symptom had adversely affected their normal activities. Disability scores (WHODAS-12 total and individual domains) increased as breathlessness worsened. To conclude, in India, conservative estimates indicate 626 million people live with breathlessness of whom 52 million people live with severe breathlessness. The symptom is associated with poorer health-related quality of life and marked disability, including reduced ability to perform daily activities.

## Introduction

Living with the symptom of breathlessness, is one of the most debilitating experiences for people with chronic complex or chronic progressive illnesses [1, 2]. Breathlessness is associated with poorer quality of life, increased anxiety and depression, worsening function and sexual wellbeing, limited activities of daily living, reduced workforce participation and increased social isolation [3–9]. The symptom also has strong negative effects on caregivers and families [10]. Healthcare systems are also affected due to frequent unplanned contact (primary and emergency care) and longer inpatient care [11, 12]. In high income countries (HICs), approximately 10% of adults live with the symptom, with prevalence rates increasing with age, advanced disease and at the end of life [13–15].

In the context of global health, estimates of the prevalence, severity and impacts from breathlessness in low- and middle-income countries (LMICs) are important, but to date have only included adults over the age of 40 [16]. LMICs account for the highest global burden of illnesses associated with breathlessness (e.g., over 90% of people with chronic obstructive pulmonary disease (COPD) live in LMICs [17, 18], with respiratory diseases being the most frequently attributed cause of breathlessness) [19]. Behavioural, workplace and environmental risk factors [20–23], and lack of access to affordable health care [20, 24] are also likely to contribute to prevalence of breathlessness in these settings [25]. As such, breathlessness is likely more frequent and impactful in LMICs, but potentially less visible. Delineating the prevalence and intensity of the symptom of breathlessness and its impacts is critical to help optimise its recognition, and effective assessment and management in these settings.

Much of the research to date in both HICs and LMICs has focused on measuring the *impact of disease* rather than *symptom*. The limited available evidence on prevalent symptoms in people with chronic complex or chronic progressive conditions in LMICs, derived mostly from qualitative studies, indicates that breathlessness presents complex and manifold challenges for the person’s physical activity, social interaction with family and friends, quality of life, income (e.g., through inability to work, having to change work or reduce hours worked) and financial burden (e.g., transportation costs to/from health facilities) [20]. Its management may be compromised by poor access to healthcare (even when this may be available), resources and health professionals’ knowledge and beliefs [21, 26]. Thus, population studies that engage with community-dwelling people living with this symptom, independently of healthcare contact, are important to help improve the understanding and evidence base for the quantification and impacts of breathlessness in LMICs. In this respect, population studies that assess *breathlessness limiting exertion* (henceforth ‘breathlessness’) using the modified Medical Research Council (mMRC) breathlessness scale [27] as a critical first step.

In this proof-of-concept study, we chose India, a lower middle-income country (LMIC), as an exemplar to explore the prevalence and impacts of breathlessness in a LMIC setting because of its:

- high burden of non-communicable diseases (61.8%) [23] including high prevalence of chronic diseases associated with breathlessness, with COPD indicated in 7% (84.8 million), [28] lung cancer in 5.4% (65.4 million), [29] and cardiovascular diseases in 4.5% (54.4 million) [30] of the population; plus high incidence of tuberculosis (0.21%; 2.95million); [31] and
- high rates of smoking (28.6% of adults) [29].

These factors may generate high prevalence rates of breathlessness. India’s high access and usage of internet and mobile technology also means that the symptom and its effects could be investigated at the population level, independently of healthcare contact or utilisation, using web-based approaches that are effective in exploring large-scale health issues [32–34].

Accordingly, the aim of this study was to explore the prevalence, severity, self-attributed underlying conditions and impacts of breathlessness across diverse aspects of personhood in community-dwelling adults in India.

## Methods

### Design

This was a cross-sectional online survey; questions were defined by the research team and then distributed by a marketing research company (Qualtrics) to adults (≥18 years) in India. Recruitment was not restricted to any state or territory.

A pre-planned convenience sample of 3000 respondents was recruited in line with previous similar population-based surveys in high-income countries, [5] stratified by age, sex and rurality using quotas for each demographic sub-group as identified in the 2011 Indian National Census.

Respondents were invited from the market research company’s double opt-in database of registered, consenting members (n = 9,875,795; of whom 6,559,326 members belong to English-only panels and 3,316,469 members belong to panels that do not report on language ability). Email invitations with a unique survey link were sent to a random sample of registered panel members selected from multiple sources to create a blended sample, reducing the risk of selection bias. The panels meet strict ISO certification requirements, [35, 36] and use rigorous quality screening of potential respondents to ensure their validity [37].

The survey remained open until the required number of respondents for each demographic cell quota completed the survey. Respondents received a financial incentive (USD $2.00–3.50) for their time and participation, with the panels ensuring incentives were competitive and equitable between communities. The survey took approximately 10–15 minutes to complete and was undertaken using a mobile phone, desktop, laptop or tablet.

A Participant Information Sheet with study details was made available to each potential respondent before commencement. Respondents were only able to join the survey after registering their informed consent to participate in the survey and for their data to be used in future research in any de-identified, aggregated form.

### Survey development and piloting

The online survey was conducted in English. Measures implemented to ensure that the survey’s content was culturally sensitive and relevant for the population of India included: using validated tools, where possible; constructing questions contextualised to India informed by resident clinical researchers; and piloting the survey questions with randomly selected community members in Mumbai, India to ensure the questions’ feasibility and cognitive interpretation.

The survey was initially piloted with 50 respondents and based on respondent feedback, changes were made to the survey to improve its usability and data accuracy. The pilot was conducted on 23 January, 2023; the survey was conducted between 1–27 February, 2023.

### Setting

India is the world’s most populous country, with over 1.4 billion people (36% of whom live in urban areas) [38]. Hindi is the most widely spoken language (57.1% of the population) [39]. English is spoken by approximately 11% of the population (~130 million), the majority of whom speak it as their second or third language [39]. As of 2018, the total literacy rate stands as 74% (82% for males; 66% for females) [40], with significant variation across the country [41]. Internet and mobile usage are sharply rising. As of 2022, 49% of the population used the internet; [42] 1.15 billion cellular mobile connections exist, and it is estimated that mobile connections in India are equivalent to 77% of the country’s population as of January 2023 [42, 43].

### Participants

Study participants were English-speaking members of the general population of India, who were registered members of an online panel provider (Qualtrics). All adults who consented to complete the survey were eligible to participate.

Potential participants self-selected to respond to an email invitation for the survey which deliberately did not mention ‘breathlessness’ but referred to ‘questions about health’ (with approval of the ethics committees).

### Measures

Self-reported demographics included age; sex; place of residence (urban vs rural) and highest level of education.

### Assessment of breathlessness limiting exertion

The presence and severity of breathlessness was assessed using the 5-point ordinal modified Medical Research Council (mMRC) breathlessness scale [27]. The mMRC scale measures the level of exertion before breathlessness limits the respondent’s function, with higher scores indicating worse functional impact. The mMRC was designed for population surveys and has been extensively used in epidemiological and clinical studies [2]. For the purposes of the current analyses, *breathlessness limiting exertion* (‘breathlessness’) was defined as mMRC ≥1 *(‘I get short of breath when hurrying on the level or walking up a slight hill’*, or worse). In the survey, a timeframe for the duration of breathlessness was not specified as per the definition of chronic breathlessness [44], nor was breathlessness defined for the respondents.

Respondents who indicated breathlessness of mMRC ≥1 were asked to indicate:

1. For how long they had experienced: a) that level of breathlessness (months/years); and b) any level of breathlessness (months/years); *and*
2. The primary condition to which they attributed their breathlessness (one response allowed from a multiple-choice list, with a free text option); *and*
3. To what degree breathlessness affected their normal activities of daily living (‘a lot’; ‘a little’; ‘not at all’) using the London Chest Activities of Daily Living (LCADL) question [45].

### Assessment of overall health and wellbeing

Health-related quality of life was assessed using the descriptive and visual analogue scale of the EuroQol five dimensions, five level (EQ-5D-5L) instrument. The five dimensions include mobility, self-care, usual activity, pain/discomfort and anxiety/depression, with five levels of severity (no problem, slight, moderate, severe and extreme problems). The EQ-5D-5L visual analogue scale (EQ-VAS) ranges from 0 to 100, where 0 and 100 represent the worst and best imaginable health states, respectively.

Disability was assessed using the World Health Organisation’s Disability Assessment Schedule (WHODAS) 2.0 12-item measure [46]. The WHODAS is a generic assessment tool for health and disability, standardised across conditions, populations and cultures. It assesses levels of functioning in six domains of life (*cognition*, *mobility*, *self-care*, *getting along*, *life activities* and *participation*), with higher scores indicating greater disability (total score range 0–48 [no disability–complete disability]; individual domain scores range 0–8 [no disability–complete disability]).

The survey questions can be accessed at https://osf.io/c7rbe/.

### Statistical analysis

Statistical analyses were conducted by a biostatistician (S.C.) and performed using the Statistical Package for the Social Sciences (SPSS) software, V28.0 (IBM Corporation, Armonk, NY; 2016). Demographic characteristics, including education, smoking, duration, and attributed cause of breathlessness were tabulated, and frequency distributions calculated for categorical variables; differences between proportions were evaluated using the Chi-square test.

We employed sample weighting (drawing upon data from the 2011 Census of India)as a technique to rectify disparities in the demographic characteristics within our sample and to enhance its alignment with the broader Indian population. We constrained our focus to the total adult population, who possessed literacy skills, as our sample was derived from respondents to an online survey. Only records within our sample that featured complete demographic data relevant to weight generation were considered. The demographic covariates included in the construction of our survey weights encompassed age, rurality and educational attainment. The variable of sex was deliberately omitted from this weighting process, as our sample’s distribution closely mirrored that of the general population in India.

Within the statistical raking process, we assigned higher weights to under-represented and lower weights to over-represented characteristics in our sample relative to their prevalence in the target population. Comprehensive demographic characteristics in both the pre-weighted and post-weighted samples are available in the Supplementary Materials (S5–S6 Tables).

Subsequently, these study-specific weights were applied to ensure the harmonisation of our study characteristics with their corresponding distributions within the broader general population, thus facilitating a more precise and accurate representation in our analysis.

No data were imputed. Ethics approval was obtained from the University of Wollongong Human Research Ethics Committee (2022/147) and the Bhatia Hospital Medical Research Society Ethics Committee (ECR/388/Inst/MH/2013/RR-19). The study’s reporting follows the STrengthening the Reporting of Observational studies in Epidemiology (STROBE) guidelines [47]. Additional information regarding the ethical, cultural, and scientific considerations specific to inclusivity in global research is included in the Supporting Information (S1 Checklist).

## Results

The study reports the weighted data. The unweighted data are provided in the Supplementary Materials (S1–S4 Tables).

### Demographic characteristics

Included respondents (n = 3,046) had a mean age of 38 years (SD 15); 57% were male, 59% lived in rural areas and 33% had completed 12^th^ grade. (Table 1) Just over one half reported being non-smokers (51%), while 47% reported a history of smoking; 50% of respondents had been exposed to second-hand smoking in the household.

**Table 1:** Characteristics of respondents (n = 3,046) by level of breathlessness measured on the modified Medical Research (mMRC) scale [weighted data].

	mMRC n (%)					Total (n = 3,046)
	0 n = 1,695 (55.7%)	1 n = 730 (24.0%)	2 n = 361 (11.8%)	3 n = 148 (4.9%)	4 n = 112 (3.7%)	
Age						
Mean (SD)	37.2 (14.4)	38.1 (14.8)	39.9 (13.8)	41.2 (16.7)	38.1 (10.2)	38.05 (14.49)
Median (min, max)	35.0 (18.0, 90.0)	35.0 (18.0, 89.0)	38.0 (18.0, 71.0)	37.0 (18.0, 69.0)	41.0 (19.0, 55.0)	35.00 (18.00, 90.00)
Sex						
Male	1032 (60.9)	399 (54.7)	182 (50.4)	101 (68.2)	28 (25.2)	1743 (57.2)
Female	663 (39.1)	331 (45.3)	177 (49.0)	47 (31.8)	83 (74.8)	1301 (42.7)
Other	0 (0)	0 (0)	2 (0.6)	0 (0)	0 (0)	2 (0.1)
Place of residence						
Urban	800 (47.2)	283 (38.8)	95 (26.3)	36 (24.3)	29 (25.9)	1243 (40.8)
Rural	895 (52.8)	447 (61.2)	266 (73.7)	112 (75.7)	83 (72.1)	1803 (59.2)
Education						
No formal education	115 (6.8)	38 (5.2)	4 (1.1)	0 (0)	22 (19.6)	179 (5.9)
Less than 10th grade	602 (35.5)	181 (24.8)	69 (19.1)	69 (46.6)	18 (16.1)	939 (30.8)
10th grade completed	245 (14.5)	98 (13.4)	119 (33.0)	8 (5.4)	60 (53.6)	530 (17.4)
12th grade completed^#^	540 (31.9)	280 (38.4)	108 (29.9)	56 (37.8)	8 (7.1)	992 (32.6)
College/University completed	107 (6.3)	67 (9.2)	31 (8.6)	8 (5.4)	2 (1.8)	215 (7.1)
Postgraduate degree completed	86 (5.1)	66 (9.0)	30 (8.3)	7 (4.7)	2 (1.8)	191 (6.3)
Smoking status						
Current smoker	372 (21.9)	141 (19.3)	129 (35.7)	86 (58.1)	21 (18.8)	749 (24.6)
Former smoker	275 (16.2)	219 (30.0)	101 (28.0)	26 (17.6)	58 (51.8)	679 (22.3)
Never smoked	1004 (59.2)	360 (49.3)	129 (35.7)	35 (23.6)	32 (28.6)	1560 (51.2)
Prefer not to say	44 (2.6)	10 (1.4)	2 (0.6)	1 (0.7)	1 (0.9)	58 (1.9)
Does anyone in your household currently smoke						
Yes	709 (41.8)	422 (57.8)	246 (68.1)	54 (36.5)	2 (1.8)	1512 (49.6)
No	986 (58.2)	308 (42.2)	115 (31.9)	94 (63.5)	30 (26.8)	1533 (50.3)
Breathlessness duration (in years)*						
Current level—Mean (SD); Median (min, max)		4.46 (5.1); 2.67 (0.1, 40.0)	3.24 (2.6); 3 (0.1, 39.3)	1.51 (2.4); 1.17 (0.1, 26.1)	2.16 (0.8); 2.08 (0.2, 10.2)	3.62 (4.2); 2.42 (0.1, 40.0)
Any level—Mean (SD); Median (min, max)		4.21 (5.3); 2.42 (0.1, 40.0)	3.46 (3.0); 3 (0.1, 34.4)	1.58 (2.6); 1.17 (0.1, 28.1)	2.47 (0.8); 3.08 (0.2, 10.2)	3.58 (4.4); 2.27 (0.1, 40.0)
Underlying primary condition*						
Poor nutrition		201 (27.5)	80 (22.2)	72 (48.6)	23 (20.5)	376 (27.8)
Other lung conditions (e.g. emphysema, bronchitis, asthma, bronchiectasis)		133 (18.2)	76 (21.1)	22 (14.9)	0 (0.0)	231 (17.1)
Anaemia		117 (16.0)	37 (10.2)	13 (8.8)	7 (6.3)	174 (12.9)
Do not know		90 (12.3)	39 (10.8)	13 (8.8)	19 (17.0)	161 (11.9)
Heart conditions		36 (4.9)	44 (12.2)	13 (8.8)	58 (51.8)	151 (11.2)
COVID		74 (10.1)	12 (3.3)	2 (1.4)	1 (0.9)	89 (6.6)
Tuberculosis		18 (2.5)	34 (9.4)	0 (0.0)	3 (2.7)	55 (4.1)
Disorders of the nerves or muscles		25 (3.4)	22 (6.1)	6 (4.1)	0 (0.0)	53 (3.9)
Other		15 (2.1)	9 (2.5)	6 (4.1)	1 (0.9)	31 (2.3)
HIV or AIDS		21 (2.9)	8 (2.2)	0 (0.0)	0 (0.0)	29 (2.1)
Cancer		0 (0.0)	0 (0.0)	1 (0.7)	0 (0.0)	1 (0.1)

*Questions on duration and underlying condition of breathlessness apply to mMRC ≥1 only.

^#^12^th^ grade is the final year of secondary/high school, with students usually aged 16–18 years.

### Breathlessness

*Breathlessness* (mMRC ≥1) was reported by 44% (1351/3046; Table 1). A larger proportion of women reported being breathless compared to men (*X*^*2*^ (1, *N* = 2983) = 32.3, *p* < 0.0001). Breathlessness was most frequently attributed to poor nutrition (28%), lung conditions other than tuberculosis (17%) or anaemia (13%). Approximately 12% of respondents did not know what was causing their breathlessness, and 4% attributed the symptom to tuberculosis. Mean duration of any level of breathlessness (mMRC 1–4) experienced was 3.6 years (SD 4.2).

Most severe breathlessness (mMRC 4) was reported by approximately 4% (112/3046), attributed to a heart condition (52%) or poor nutrition (21%). None attributed it to a respiratory condition other than tuberculosis, while 17% did not know the reason for their symptom.

#### Impacts of breathlessness

**Fig 1:**
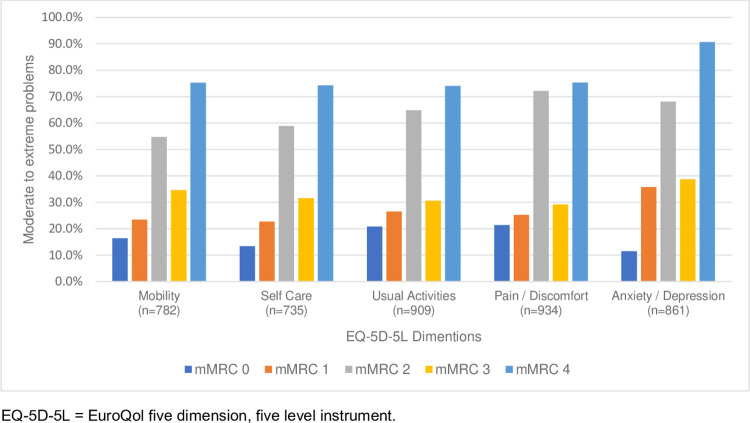
Proportion of moderate-to-extreme problems by quality of life (EQ-5D-5L) dimensions and level of breathlessness (modified Medical Research [mMRC] breathlessness scale) reported by 3,046 community-dwelling adults in an online survey for India [weighted data].

*Health-related quality of life*. Compared to people without breathlessness, higher proportions of those with breathlessness (mMRC ≥1) reported problems across all EQ-5D-5L five dimensions (Table 2), including moderate-to-severe problems (Fig 1). Every grade within mMRC greater than 0 had higher (worse) scores for quality of life. (Table 2 and Fig 1) People whose activity was more limited by breathlessness (mMRC 4) consistently reported the highest rates of extreme problems.

**Table 2:** Quality of Life (EQ-5D-5L) by level of breathlessness (measured on the modified Medical Research Council [mMRC] breathlessness scale) for 3,046 respondents to an online survey in India [weighted data].

	mMRC n (%)					Total (n = 3,046)
	0 n = 1,695 (55.7%)	1 n = 730 (24.0%)	2 n = 361 (11.8%)	3 n = 148 (4.9%)	4 n = 112 (3.7%)	
**Mobility**						
I have no problems in walking about	1230 (72.6)	361 (49.5)	86 (23.8)	25 (16.9)	27 (24.1)	1729 (56.8)
I have slight problems in walking about	187 (11.0)	198 (27.1)	78 (21.6)	72 (48.6)	0 (0)	535 (17.6)
I have moderate problems in walking about	129 (7.6)	85 (11.6)	141 (39.1)	30 (20.3)	0 (0)	385 (12.6)
I have severe problems in walking about	120 (7.1)	68 (9.3)	44 (12.2)	19 (12.8)	0 (0)	251 (8.2)
I am unable to walk about	29 (1.7)	18 (2.5)	12 (3.3)	2 (1.4)	85 (75.9)	146 (4.8)
**Self-care**						
I have no problems washing or dressing myself	1230 (72.6)	385 (52.7)	78 (21.6)	35 (23.6)	28 (25.0)	1756 (57.6)
I have slight problems washing or dressing myself	239 (14.1)	179 (24.5)	71 (19.7)	67 (45.3)	0 (0)	556 (18.3)
I have moderate problems washing or dressing myself	110 (6.5)	136 (18.6)	166 (46)	10 (6.8)	0 (0)	422 (13.9)
I have severe problems washing or dressing myself	69 (4.1)	29 (4.0)	30 (8.3)	33 (22.3)	5 (4.5)	166 (5.4)
I am unable to wash or dress myself	47 (2.8)	1 (0.1)	16 (4.4)	3 (2.0)	79 (70.5)	146 (4.8)
**Usual Activity**						
I have no problems doing my usual activities	1026 (60.5)	317 (43.4)	72 (19.9)	80 (54.1)	28 (25.0)	1523 (50.0)
I have slight problems doing my usual activities	316 (18.6)	219 (30.0)	54 (15.0)	22 (14.9)	1 (0.9)	612 (20.1)
I have moderate problems doing my usual activities	323 (19.1)	148 (20.3)	193 (53.5)	20 (13.5)	0 (0)	684 (22.5)
I have severe problems doing my usual activities	8 (0.5)	43 (5.9)	32 (8.9)	22 (14.9)	11 (9.8)	116 (3.8)
I am unable to do my usual activities	22 (1.3)	3 (0.4)	10 (2.8)	4 (2.7)	72 (64.3)	111 (3.6)
**Pain/Discomfort**						
I have no pain or discomfort	803 (47.4)	164 (22.5)	52 (14.4)	19 (12.8)	22 (19.6)	1060 (34.8)
I have slight pain or discomfort	530 (31.3)	382 (52.3)	49 (13.6)	86 (58.1)	6 (5.4)	1053 (34.6)
I have moderate pain or discomfort	280 (16.5)	100 (13.7)	184 (51)	14 (9.5)	1 (0.9)	579 (19.0)
I have severe pain or discomfort	25 (1.5)	82 (11.2)	65 (18.0)	27 (18.2)	3 (2.7)	202 (6.6)
I have extreme pain or discomfort	57 (3.4)	2 (0.3)	11 (3.0)	2 (1.4)	80 (71.4)	152 (5.0)
**Anxiety / Depression**						
I am not anxious or depressed	944 (55.7)	167 (22.9)	56 (15.5)	80 (54.1)	5 (4.5)	1252 (41.1)
I am slightly anxious or depressed	556 (32.8)	301 (41.2)	59 (16.3)	10 (6.8)	5 (4.5)	931 (30.6)
I am moderately anxious or depressed	85 (5.0)	155 (21.2)	170 (47.1)	23 (15.5)	0 (0)	433 (14.2)
I am severely anxious or depressed	63 (3.7)	70 (9.6)	61 (16.9)	31 (20.9)	10 (8.9)	235 (7.7)
I am extremely anxious or depressed	47 (2.8)	37 (5.1)	15 (4.2)	4 (2.7)	92 (82.1)	195 (6.4)
EQ-VAS score—M (SD)	82.2 (18.7)	70.6 (20.9)	72.0 (18.0)	72.1 (20.5)	90.6 (7.0)	78.0 (19.9)

The mean EQ-VAS score was 78 (SD 19.9) for the whole population. (Table 2) Surprisingly, better perceived health was reported as breathlessness increased, with people with most severe breathlessness reporting having best perceived health (EQ-VAS mean score 91 [SD 7]).

*Activities of daily living*. Of the people who reported breathlessness, 81% (1092/1351) indicated the symptom had adversely affected their normal activities to some degree (‘a little/lot’; Table 3), increasing to 98% for those reporting most severe breathlessness. People with mMRC 4 were most likely be impacted by their breathlessness compared to those with mMRC 1–3 (*X*^*2*^ (1, *N* = 1351) = 23.8, *p* < 0.0001).

**Table 3:** Impact of breathlessness measured on the modified Medical Research Council (mMRC ≥1; n = 1,351) on respondents’ everyday activities reported in an online survey for India [weighted data].

	mMRC n (%)				Total (n = 1,351)
	1 n = 730 (24.0%)	2 n = 361 (11.8%)	3 n = 148 (4.9%)	4 n = 112 (3.7%)	
Degree to which breathlessness affects a person’s normal activities of daily life					
A lot	118 (16.2)	122 (33.8)	34 (23.0)	79 (70.5)	353 (26.1)
A little	471 (64.5)	194 (53.7)	43 (29.1)	31 (27.7)	739 (54.7)
Not at all	141 (19.3)	45 (12.5)	71 (48.0)	2 (1.8)	259 (19.2)

**Fig 2:**
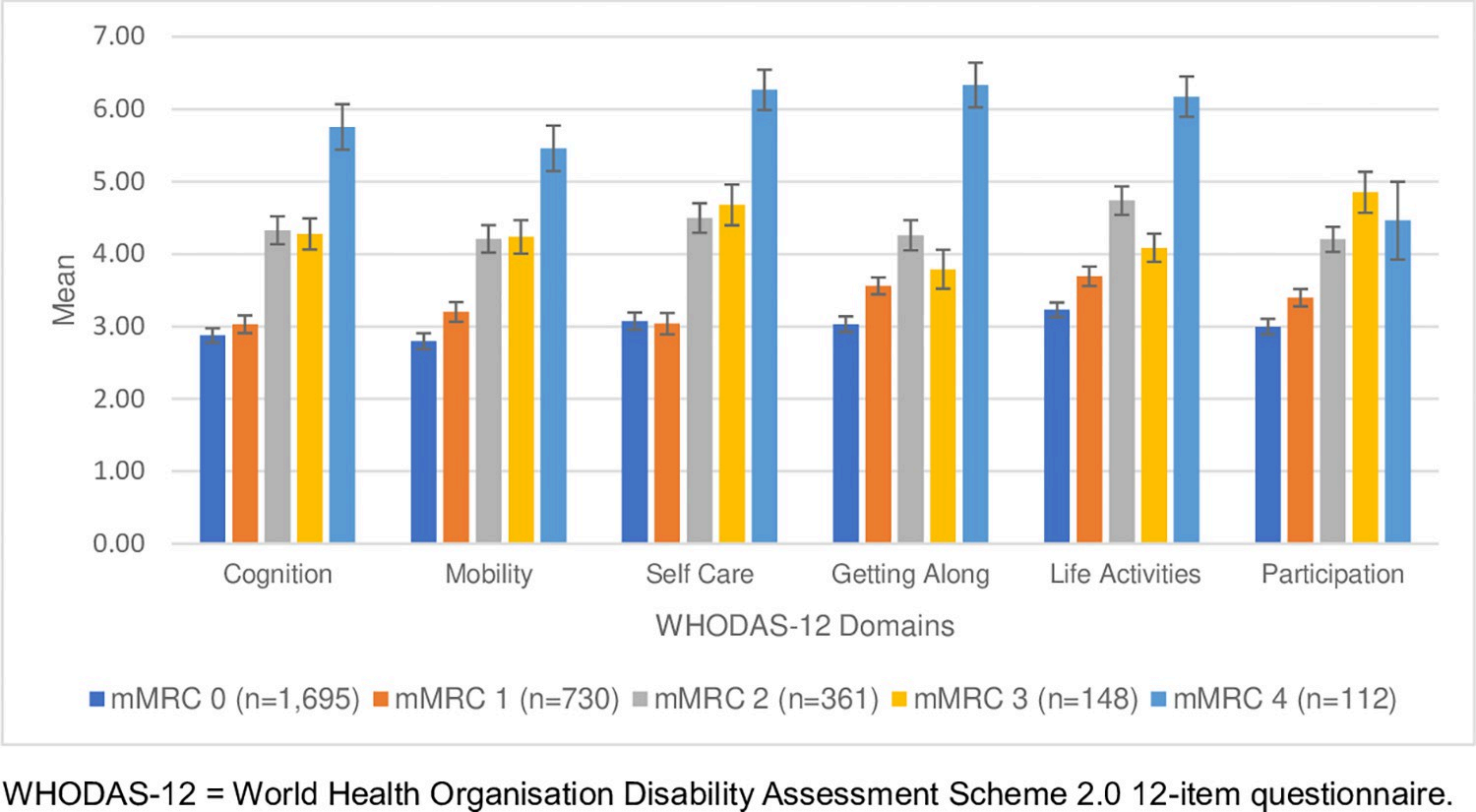
Mean disability scores with 95% CIs for WHODAS-12 individual domains and level of breathlessness (modified Medical Research Council [mMRC] breathlessness scale) reported by 3,046 community-dwelling adults in an online survey for India [weighted data].

*Disability*. The total disability (WHODAS-12) mean score was 20 (SD 11; Table 4). The most compromised disability domains for those reporting breathlessness were *Life activities* (mMRC 1–2), *Participation* (mMRC 3) and *Getting along* (mMRC 4). Disability scores (total and for each of the six domains) increased as breathlessness became more severe. (Table 4 and Fig 2) People with breathlessness experienced disability for 10 days per month, including reducing or being unable to perform their usual activities or work for 3 and 4 days per month, respectively. (Table 4) Those with mMRC 4 experienced disability for 10 days per month, including reducing and completely ceasing their usual activities or work for 3 and 7 days per month, respectively.

**Table 4:** Weighted Mean (SD) and median (IQR) of the World Health Organisation Disability Assessment Schedule 2.0 12-item (WHODAS-12) and its individual domains and breathlessness measured on the modified Medical Research Council (mMRC) breathlessness scale for 3,046 respondents to an online survey in India [weighted data].

	mMRC n (%)					Total (n = 3,046)
	0 n = 1,695 (55.7%)	1 n = 730 (24.0%)	2 n = 361 (11.8%)	3 n = 148 (4.9%)	4 n = 112 (3.7%)	
**WHODAS Total score—M(SD); Me (min,max)**	18 (11.5); 19 (0, 48)	19.9 (7.8); 21 (0, 46)	26.2 (8.9); 25 (0, 48)	25.9 (6.9); 28 (0, 44)	34.4 (8.8); 34 (4, 48)	20.4 (10.9); 21 (0, 48)
1 –Cognition	2.9 (2.1); 3 (0, 8)	3 (1.7); 3 (0, 8)	4.3 (1.9); 4 (0, 8)	4.3 (1.3); 4 (0, 8)	5.8 (1.7); 6 (0, 8)	3.3 (2.1); 3 (0, 8)
2 –Mobility	2.8 (2.3); 3 (0, 8)	3.2 (1.9); 3 (0, 8)	4.2 (1.8); 4 (0, 8)	4.2 (1.4); 5 (0, 8)	5.5 (1.7); 5 (0, 8)	3.2 (2.2); 3 (0, 8)
3 –Self Care	3.1 (2.5); 3 (0, 8)	3 (2); 3 (0, 8)	4.5 (2); 5 (0, 8)	4.7 (1.7); 5 (0, 8)	6.3 (1.5); 6 (0, 8)	3.4 (2.4); 4 (0, 8)
4 –Getting along	3 (2.3); 2 (0, 8)	3.6 (1.6); 4 (0, 8)	4.3 (2); 4 (0, 8)	3.8 (1.7); 3 (0, 8)	6.3 (1.6); 6 (0, 8)	3.5 (2.2); 3 (0, 8)
5 –Life Activities	3.2 (2.1); 3 (0, 8)	3.7 (1.8); 4 (0, 8)	4.7 (1.9); 5 (0, 8)	4.1 (1.2); 4 (0, 8)	6.2 (1.5); 6 (0, 8)	3.7 (2.1); 4 (0, 8)
6 –Participation	3 (2.3); 3 (0, 8)	3.4 (1.6); 4 (0, 8)	4.2 (1.7); 4 (0, 8)	4.9 (1.7); 6 (0, 8)	4.5 (2.9); 5 (0, 8)	3.4 (2.1); 3 (0, 8)
Overall, in the past 30 days, how many days were these difficulties present M(SD); Me (Min,Max)	14 (9.2); 12 (1, 30)	13.9 (7.1); 12 (1, 30)	7.6 (7.1); 4 (1, 30)	10.8 (6.9); 10 (2, 30)	12.7 (8.9); 10 (1, 30)	12.4 (9.1); 10 (1, 30)
In the past 30 days, for how many days were you totally unable to carry out your usual activities or work because of any health condition? M(SD); Me (Min,Max)	7.2 (7); 5 (0, 27)	7.5 (5.4); 7 (0, 29)	3.3 (4.2); 3 (0, 26)	5 (2.7); 7 (0, 29)	6.4 (6.9); 4 (0, 29)	6.2 (7.3); 4 (0, 29)
In the past 30 days, not counting the days that you were totally unable, for how many days did you cut back or reduce your usual activities or work because of any health condition? M(SD); Me (Min,Max)	5.5 (5.1); 5 (0, 29)	6.2 (4.8); 5 (0, 29)	3.2 (3.9); 3 (0, 23)	2.4 (2.1); 3 (0, 29)	4.6 (4.9); 3 (0, 29)	4.2 (4.9); 3 (0, 29)

## Discussion

### Key findings

This first-of-its-kind investigation found a high prevalence of breathlessness in community-dwelling adults in India, with 44% of the population experiencing any breathlessness (mMRC ≥1) and almost 4% experiencing most severe breathlessness (mMRC 4). At the population level in India, this translates to 626 million people living with breathlessness of some degree of intensity and 52 million people living with such debilitating breathlessness that they are housebound or have difficulty dressing or undressing because of it. Findings also indicate that breathlessness for the Indian population is associated with poorer quality of life and significant disability, including reduced ability to undertake activities of daily living.

The prevalence rates reported for any breathlessness in India in this study were higher than some of the limited data available for HICs. Australian population studies using similar methodology have reported prevalence rates of mMRC ≥1 of approximately 40% (n = 10,072) [13] and 42% (n = 10,033), [48] while a US study (n = 10,881) has reported breathlessness rates of 22% for mMRC ≥1 [49]. The exception is a UK online survey (n = 356,799) where 71% of adults reported breathlessness mMRC ≥1 [50].

Self-attributed causes for breathlessness in India included conditions commonly associated with breathlessness (e.g., respiratory or heart conditions) [19], but also factors not identified to date in other settings (e.g., malnourishment or anaemia). Of note, respiratory conditions (other than tuberculosis) were not indicated as a factor in those reporting most severe breathlessness. One in nine people (~12%) could not attribute a cause for their symptom (increasing to 17% for those with most severe breathlessness), potentially indicating a gap in health-seeking behaviour or how breathlessness is conceptualised, identified, assessed, diagnosed or managed in India. In addition to underlying aetiologies, high prevalence of risk factors associated with breathlessness (e.g., history of smoking, passive smoking, cooking with solid fuels, occupational exposure or poor air quality) may also be contributing factors for the rates of breathlessness experienced in the Indian communities. Consistent with previous studies [51, 52], a larger proportion of women in our study reported being breathless compared to men.

In line with current understanding of the impact of breathlessness, our findings also indicate that breathlessness has far-reaching consequences for individuals’ wellbeing. The findings for India, however, indicate that *quality of life* was markedly compromised, spanning physical (e.g., mobility) and psychological (e.g., anxiety) domains, with people with most severe breathlessness experiencing extreme adverse impairment. *Daily activities* were also negatively impacted by breathlessness resulting in disruption as people go about their daily life. The impact was amplified with high rates of reported *disability* that permeates all aspects of a person’s life. Of note, people with breathlessness of mMRC 3 seem to be reporting less impact than expected which might be due to their having adjusted their lifestyle in order to cope with it.

Overall, our findings are consistent with the notion that doing new things is often a formidable barrier for those living with breathlessness. Instead, people tend to focus on managing their usual or essential activities and tasks, forgoing social interaction and participation. This coping mechanism means their worlds may shrink to accommodate the most essential tasks while forgoing almost everything else. This is consistent with the construct of ‘social death’, a shrinking of societal participation, whereby people experience increasing social isolation as they cope with increasing breathlessness by forgoing social activities and decreasing social engagements [53]. Given the magnitude of the problem reported in this study, uncovering the true extent of the impact of this symptom in India would require a nuanced exploration of the societal and cultural norms and circumstances that may play a role in how the symptom is perceived in the community, as well as a fundamental shift in how the symptom is identified in routine clinical consultations.

Disability in this study was higher than previous reports for India, with a mean total score 20.4 (SD 10.9) compared to 17.4 (SD 17.2) reported for people 65 years or older [54] This is in the context of our study sample being relatively young (median age 38 years). Our findings also indicate poorer quality of life than previously reported. In a cross-sectional survey (n = 2,409) to generate an Indian EQ-5D-5L value set, using face-to-face interviews in five Indian states, more people reported ‘no problems’ and fewer reported any problem including ‘moderate to extreme problems’ compared to our study.

### Strengths and limitations

Strengths of the study include the use of quotas which reflect the total population of India and the engagement with people living with breathlessness independently of health care contact, potentially reaching people who were unable or unwilling to access clinical services for their breathlessness. However, its online delivery limited responses to those with internet access, which contributed to a greater proportion of respondents with higher education status and potentially missing people with lower socioeconomic status who may be more likely to experience long-term breathlessness.

The study has several limitations. First, all measures were self-reported and could not be clinically verified, introducing potential bias in the results. Participants were not asked about the history of any treated pulmonary tuberculosis as a potential underlying cause of breathlessness. Nutrition was also self-assessed. It is important know what is the perceived dominant underlying cause attributed by each respondent. Such information can highlight discrepancies from clinically generated attributions in subsequent research. Second, the survey was conducted in English only, limiting the sample. With more than 31 languages spoken in India [55], each with at least one million first language speakers, future studies should investigate the prevalence and impacts of breathlessness in more diverse populations, and longitudinally. The respondents’ proficiency in English was self-determined, without qualification whether that was the respondents’ first or second/third language. Future studies should explore how questions about breathlessness are understood and interpreted, including those used in validated tools such as the mMRC [56, 57]. Third, the study sample was positively skewed for *education*, which may have been due to its online delivery. Subsequently, the data were weighted without the 23% of people in India who do not have formal education.

### Implications for clinical research, practice and policy

Exploring the prevalence and impacts of breathlessness in the English-speaking population in India is an important proof-of-principle, providing preliminary information about the prevalence, severity and impacts of this symptom in the community. The study provides foundational data to compare and inform our understanding of this disabling symptom and to help inform the development of better recognition, assessment and management practices [20]. Future studies should broaden the investigation to include non-English speaking populations in India, multi-dimensional assessments of breathlessness and its underlying conditions, including health-seeking behaviour by people living with this symptom [21].

Given the multi-factorial impacts of breathlessness and the disability it causes, clinicians should actively seek to identify these issues in routine practice. Equally, future research should explore the broader financial implications of living with breathlessness long-term, including being a potential driver for poverty. The absence of a safety net when employment or ability to work is compromised might also mean that consequences of living with this symptom are greater in LMICs.

## Conclusion

This study estimates conservatively that 626 million people live with breathlessness in India of whom 52 million people may live with severe breathlessness. Breathlessness is associated with poorer quality of life and marked disability, including reduced ability to perform activities of daily living. These effects need to be investigated in larger cross-sectional and longitudinal studies with diverse populations to further delineate the impact of breathlessness at the individual, societal and health system levels, and help inform the development and delivery of targeted, person-centred symptom management and care.

## Supporting information

S1 Checklist Inclusivity in global research form. (DOCX)

S1 Table Characteristics of respondents (n = 3,046) by level of breathlessness measured on the modified Medical Research (mMRC) scale [unweighted data]. (DOCX)

S2 Table Quality of Life (EQ-5D-5L) by level of breathlessness (measured on the modified Medical Research Council [mMRC] breathlessness scale) for 3,046 respondents to an online survey in India [unweighted data]. (DOCX)

S3 Table Impact of breathlessness measured on the modified Medical Research Council (mMRC ≥1; n = 1,351) on respondents’ everyday activities reported in an online survey for India [unweighted data]. (DOCX)

S4 Table Mean (SD) and median (IQR) of the World Health Organisation Disability Assessment Schedule 2.0 12-item (WHODAS-12) and its individual domains and breathlessness measured on the modified Medical Research Council (mMRC) breathlessness scale for 3,046 respondents to an online survey in India [unweighted data]. (DOCX)

S5 Table Population and sample characteristics used for creating sampling weights. (DOCX)

S6 Table India Breathlessness Survey and 2011 Census of India Populations and variables used to create a sample weight. (DOCX)
