# Supplementary material for: Prevalence, severity and impacts of breathlessness in Indian adults: An exploratory, nationally representative, cross-sectional online survey
Source: PLOS Glob Public Health. 2024 May 2;4(5):e0002655. doi: 10.1371/journal.pgph.0002655 (PMC11065295; doi:10.1371/journal.pgph.0002655)
Supplement: S1 Checklist — (DOCX) [file pgph.0002655.s001.docx]

Inclusivity in global research

PLOS’ policy on inclusivity in global research aims to improve transparency in the reporting of research performed outside of researchers’ own country or community and ensures that PLOS publications reporting global research adhere to high standards for research ethics and authorship. Authors of relevant research articles may be asked to complete the questionnaire below, which outlines ethical, cultural, and scientific considerations specific to inclusivity in global research. This questionnaire may be requested when researchers have travelled to a different country to conduct research, if research uses samples collected in another country, research with Indigenous populations or their lands, or if research is on cultural artefacts. Researchers travelling to another country solely to use laboratory equipment will not normally be required to complete the questionnaire. However, the questionnaire can be requested at the journal’s discretion for any submission – if you have been requested to complete this questionnaire by the PLOS journal you submitted to, please do so.

Please complete the questionnaire below and include this as a Supporting Information file with your manuscript. Note that if your paper is accepted for publication, this checklist will be published with your article in the supporting information files. Please ensure that you reference the checklist in the main body of your manuscript. We suggest adding a subsection ‘Inclusivity in global research’ to your Methods section and adding the following sentence: “Additional information regarding the ethical, cultural, and scientific considerations specific to inclusivity in global research is included in the Supporting Information (SX Checklist)”

The questions have been designed to be applicable to a wide range of study types, and there are subsections for both human subjects research and non-human subjects research. If any of the questions are not relevant to your research please mark them as “N/A” as appropriate.

**Ethical considerations, permits and authorship**

*This section is applicable to all research types.*

Provide details as to who granted permissions and/or consent for the study to take place in the Methods section of your manuscript. This should include the names of **all** ethics boards, governmental organizations, community leaders or other bodies that provided approval for the study. If individuals provided approval refer to these people by their role or title but do not list their name(s).

Reported on page number: 12

If there were any deviations from the study protocol after approval was obtained please provide details of these changes in the Methods section of your manuscript.
Did this study involve local collaborators that are residents of the country where the research was conducted or members of the community studied? If you do not have any authors from said communities, please provide an explanation for this below.

Reported on page number: N/A

The joint-first and senior authors on this study are resident clinical researchers with extensive experience, knowledge and expertise in airway disease, interstitial lung disease, pulmonary hypertension, sleep-disordered breathing, advanced lung disease, tuberculosis, and palliative care. As practicing physicians in two large teaching hospitals in Mumbai, Maharashtra, India, they also provided invaluable guidance and insight into the cultural context in India and the challenges clinicians face in identifying and treating breathlessness in India.

Everyone listed as an author should meet PLOS’ criteria for authorship and all individuals who meet these criteria should be included in the author byline, rather than the acknowledgements. For further information please see the journal’s Authorship Policy.

**Human subjects research (e.g. health research, medical research, cross-cultural psychology)**

Did you obtain written informed consent from a representative of the local community or region before the research took place? How did you establish who speaks for the community? Details of written informed consent obtained from study participants should be reported separately in the Methods section of your manuscript.

The senior author and study investigator is a practicing physician and researcher at Bhatia Hospital and Medical Research Centre, Mumbai, Maharashtra, India. Therefore, local ethics approval was sought and obtained from the Bhatia Hospital Medical Research Society Ethics Committee (ECR/388/Inst/MH/2013/RR-19).

There was a twofold consent process: firstly to join the Qualtrics panel and secondly to consent to this specific study. For the specific study, informed consent was obtained from all study participants. A Participant Information Sheet detailing the study was made available to all potential respondents before study commencement. Respondents were only able to join the survey after registering their informed consent to participate in the survey *and* for their data to be used in future research in any de-identified, aggregated form.

How did members of the local community provide input on the aims of the research investigation, its methodology, and its anticipated outcome(s)?

The study, including the design of its aim, methodology and outcomes, was a collaboration between local and international experts in long-term breathlessness. The entire study investigator team met regularly to discuss the design, piloting (with the changes that were made a result of the pilot), progress of data collection and plan the analysis and dissemination of results. The joint-first and senior authors with local expertise contributed to the design and development of the survey, guided the contextualizing of the survey questions to ensure they are culturally sensitive and relevant for the population of India, and facilitated the piloting of the survey questions with a randomly selected community members in Mumbai to ensure their feasibility and cognitive interpretation.

When engaging with the local community, how did you ensure that the informed consent documents and other materials could be understood by local stakeholders?

The survey for this exploratory study was conducted only in English. There was no formal language assessment; instead, proficiency in the English language was self-determined by respondents. The survey questions were piloted with a randomly selected community members in Mumbai, India who self-identified as speakers of English, to ensure their acceptability, cognitive interpretation and feasibility. The survey was further piloted with 50 respondents online and based on feedback, changes were made to improve the survey’s usability and data accuracy before its full launch. As part of the informed consent process, a Participant Information Sheet was made available to all potential respondents before study commencement, with contact details for the Lead Investigator and ethics committee, as well as a dedicated study email address that local stakeholders could use for any study-related enquiries.

Will the findings of the research be made available in an understandable format to stakeholders in the community where the study was conducted (e.g. via a presentation, summary report, copies of publications, etc.)? Please provide details of how this will be achieved.

The study’s findings will be presented to the local clinical communities using presentations and summary reports. Open Access publications will ensure the results are freely available to the local and international research and clinical community. The websites associated with ech investigator will also be provided with an agreed lay summry of the findings.

**Non-human subjects research using specimens/ animals collected as part of the study, or those housed in archival collections. Examples include archaeology, paleontology, botany and zoology.**

Did the permission you obtained from a local authority to perform the study include an agreement on access to outputs and benefit sharing? This may include procedures to enable fair distribution of the benefits and resources arising from the research performed. Please include any details of Prior Informed Consent and Benefit Sharing Agreements obtained. These may be required by field-specific regulations, for example the Convention on Biological Diversity (CBD) and the associated Nagoya Protocol.

N/A

If the material used in your study was imported, please A) provide the year it was imported and B) indicate whether permits were obtained to import/export the materials used, C) provide details of any permits obtained. If this information is not available, please indicate this.

N/A

If you used archival specimens, please state how the material used in your study was acquired by the institute it is held in and provide details of any permits obtained for the original excavations/ sample collection. If this information is not available, please indicate this.

N/A

How was the potential cultural significance of the materials collected in your study to local communities considered in your research design? Were Indigenous peoples and/or local researchers and institutions involved with archaeological excavations / collection of specimens? If so, please provide a description of their involvement.

N/A

If your manuscript includes photographs of human remains please indicate whether authors obtained permission from descendants or affiliated cultural communities to do so.

N/A
