# Supplementary material for: Prevalence, severity and impacts of breathlessness in Indian adults: An exploratory, nationally representative, cross-sectional online survey
Source: PLOS Glob Public Health. 2024 May 2;4(5):e0002655. doi: 10.1371/journal.pgph.0002655 (PMC11065295; doi:10.1371/journal.pgph.0002655)
Supplement: S1 Table — (DOCX) [file pgph.0002655.s002.docx]

**S1 Table**  Characteristics of respondents (n=3,046) by level of breathlessness measured on the modified Medical Research (mMRC) scale [unweighted data]

|  | **mMRC**  **n (%)** | | | | | **Total**  **(n=3,046)** |
| --- | --- | --- | --- | --- | --- | --- |
|  | **0**  **1,471 (48.3)** | **1**  **939 (30.8)** | **2**  **431 (14.1)** | **3**  **140 (4.6)** | **4**  **65 (2.1)** |  |
| Age |  |  |  |  |  |  |
| Mean (SD) | 37.2 (14.4) | 38.1 (14.8) | 39.9 (13.8) | 41.2 (16.7) | 38.1 (10.2) | 38.05 (14.49) |
| Median (min, max) | 35.0  (18.0, 90.0) | 35.0  (18.0, 89.0) | 38.0  (18.0, 71.0) | 37.0  (18.0, 69.0) | 41.0  (19.0, 55.0) | 35.00  (18.00, 90.00) |
| Sex |  |  |  |  |  |  |
| Male | 920 (62.5) | 452 (48.1) | 184 (42.7) | 58 (41.4) | 21 (32.3) | 1635 (53.7) |
| Female | 550 (37.4) | 487 (51.9) | 245 (56.8) | 81 (57.9) | 44 (67.7) | 1407 (46.2) |
| Other | 0 (0) | 0 (0) | 2 (0.5) | 1 (0.7) | 0 (0) | 3 (0.1) |
| Prefer not to say | 1 (0.1) | 0 (0) | 0 (0) | 0 (0) | 0 (0) | 1 (0.0) |
| Place of residence |  |  |  |  |  |  |
| Urban | 478 (32.5) | 361 (38.4) | 146 (33.9) | 41 (29.3) | 8 (12.3) | 1034 (33.9) |
| Rural | 993 (67.5) | 578 (61.6) | 285 (66.1) | 99 (70.7) | 57 (87.7) | 2012 (66.1) |
| Education |  |  |  |  |  |  |
| No formal education | 6 (0.4) | 5 (0.5) | 1 (0.2) | 0 (0) | 1 (1.5) | 13 (0.4) |
| Less than 10th grade | 17 (1.2) | 9 (1) | 5 (1.2) | 2 (1.4) | 1 (1.5) | 34 (1.1) |
| 10th grade completed | 60 (4.1) | 24 (2.6) | 48 (11.1) | 3 (2.1) | 27 (41.5) | 162 (5.3) |
| 12th grade completed | 382 (26) | 233 (24.8) | 121 (28.1) | 55 (39.3) | 3 (4.6) | 794 (26.1) |
| College/University completed | 574 (39) | 383 (40.8) | 140 (32.5) | 45 (32.1) | 17 (26.2) | 1159 (38.0) |
| Postgraduate degree completed | 432 (29.4) | 285 (30.4) | 116 (26.9) | 35 (25) | 16 (24.6) | 884 (29.0) |
| Smoking status |  |  |  |  |  |  |
| Current smoker | 434 (29.5) | 331 (35.3) | 176 (40.8) | 55 (39.3) | 14 (21.5) | 1010 (33.2) |
| Former smoker | 204 (13.9) | 155 (16.5) | 93 (21.6) | 24 (17.1) | 30 (46.2) | 506 (16.6) |
| Never smoked | 810 (55.1) | 442 (47.1) | 157 (36.4) | 59 (42.1) | 19 (29.2) | 1487 (48.8) |
| Prefer not to say | 23 (1.6) | 11 (1.2) | 5 (1.2) | 2 (1.4) | 2 (3.1) | 43 (1.4) |
| Does anyone in your household currently smoke |  |  |  |  |  |  |
| Yes | 677 (46) | 547 (58.3) | 290 (67.3) | 87 (62.1) | 46 (70.8) | 1647 (54.1) |
| No | 794 (54) | 392 (41.7) | 141 (32.7) | 53 (37.9) | 19 (29.2) | 1399 (45.9) |
| Breathlessness duration (in years)* |  |  |  |  |  |  |
| Current level |  | 3.2 (4);  2.3 (0.1, 40) | 3.5 (4.1);  3 (0.1, 39.3) | 3 (3.1);  2.4 (0.1, 26.1) | 2.5 (1.4);  2.1 (0.2, 10.2) | 3.3 (3.9);  2.3 (0.1, 40.0) |
| Any level |  | 3.3 (4.2);  2.2 (0.1, 40) | 3.5 (4.1);  2.9 (0.1, 34.4) | 3.1 (3.6);  2.5 (0.1, 28.1) | 2.5 (1.5);  2.1 (0.2, 10.2) | 3.3 (4.1);  2.3 (0.1, 40.0) |
| Underlying primary condition* |  |  |  |  |  |  |
| Other lung conditions (e.g. emphysema, bronchitis, asthma, bronchiectasis) |  | 252 (26.8) | 76 (17.6) | 21 (15.0) | 1 (1.5) | 350 (22.2) |
| Poor nutrition |  | 207 (22) | 96 (22.3) | 30 (21.4) | 6 (9.2) | 339 (21.5) |
| Anaemia |  | 95 (10.1) | 59 (13.7) | 34 (24.3) | 3 (4.6) | 191 (12.1) |
| Heart conditions |  | 84 (8.9) | 59 (13.7) | 12 (8.6) | 29 (44.6) | 184 (11.7) |
| Do not know |  | 116 (12.4) | 35 (8.1) | 12 (8.6) | 8 (12.3) | 171 (10.9) |
| COVID |  | 65 (6.9) | 20 (4.6) | 7 (5.0) | 4 (6.2) | 96 (6.1) |
| Other |  | 57 (6.1) | 12 (2.8) | 8 (5.7) | 3 (4.6) | 80 (5.1) |
| Tuberculosis |  | 19 (2.0) | 40 (9.3) | 5 (3.6) | 9 (13.8) | 73 (4.6) |
| Disorders of the nerves or muscles |  | 33 (3.5) | 25 (5.8) | 10 (7.1) | 1 (1.5) | 69 (4.4) |
| HIV or AIDS |  | 9 (1) | 6 (1.4) | 0 (0) | 0 (0) | 15 (1) |
| Cancer |  | 2 (0.2) | 3 (0.7) | 1 (0.7) | 1 (1.5) | 7 (0.4) |

*Questions on duration and underlying condition of breathlessness apply to mMRC ≥1 only
