# Supplementary material for: Prevalence, severity and impacts of breathlessness in Indian adults: An exploratory, nationally representative, cross-sectional online survey
Source: PLOS Glob Public Health. 2024 May 2;4(5):e0002655. doi: 10.1371/journal.pgph.0002655 (PMC11065295; doi:10.1371/journal.pgph.0002655)
Supplement: S2 Table — (DOCX) [file pgph.0002655.s003.docx]

**S2 Table**  Quality of Life (EQ-5D-5L) by level of breathlessness (measured on the modified Medical Research Council [mMRC] breathlessness scale) for 3,046 respondents to an online survey in India [unweighted data].

|  | **mMRC**  **n (%)** | | | | | **Total**  **(n=3,046)** |
| --- | --- | --- | --- | --- | --- | --- |
|  | **0**  **1,471 (48.3)** | **1**  **939 (30.8)** | **2**  **431 (14.1)** | **3**  **140 (4.6)** | **4**  **65 (2.1)** |  |
| **Mobility** | | | | | | |
| I have no problems in walking about | 1115 (75.8) | 410 (43.7) | 85 (19.7) | 43 (30.7) | 16 (24.6) | 1669 (54.8) |
| I have slight problems in walking about | 204 (13.9) | 380 (40.5) | 110 (25.5) | 28 (20) | 3 (4.6) | 725 (23.8) |
| I have moderate problems in walking about | 98 (6.7) | 115 (12.2) | 153 (35.5) | 24 (17.1) | 4 (6.2) | 394 (12.9) |
| I have severe problems in walking about | 27 (1.8) | 32 (3.4) | 67 (15.5) | 37 (26.4) | 2 (3.1) | 165 (5.4) |
| I am unable to walk about | 27 (1.8) | 2 (0.2) | 16 (3.7) | 8 (5.7) | 40 (61.5) | 93 (3.1) |
| **Self-care** | | | | | | |
| I have no problems washing or dressing myself | 1181 (80.3) | 510 (54.3) | 97 (22.5) | 51 (36.4) | 19 (29.2) | 1858 (61.0) |
| I have slight problems washing or dressing myself | 154 (10.5) | 291 (31) | 84 (19.5) | 24 (17.1) | 2 (3.1) | 555 (18.2) |
| I have moderate problems washing or dressing myself | 85 (5.8) | 106 (11.3) | 157 (36.4) | 32 (22.9) | 3 (4.6) | 383 (12.6) |
| I have severe problems washing or dressing myself | 25 (1.7) | 30 (3.2) | 70 (16.2) | 27 (19.3) | 5 (7.7) | 157 (5.2) |
| I am unable to wash or dress myself | 26 (1.8) | 2 (0.2) | 23 (5.3) | 6 (4.3) | 36 (55.4) | 93 (3.1) |
| **Usual Activity** | | | | | | |
| I have no problems doing my usual activities | 944 (64.2) | 364 (38.8) | 77 (17.9) | 46 (32.9) | 18 (27.7) | 1449 (47.6) |
| I have slight problems doing my usual activities | 379 (25.8) | 392 (41.7) | 111 (25.8) | 27 (19.3) | 5 (7.7) | 914 (30.0) |
| I have moderate problems doing my usual activities | 106 (7.2) | 149 (15.9) | 169 (39.2) | 25 (17.9) | 2 (3.1) | 451 (14.8) |
| I have severe problems doing my usual activities | 21 (1.4) | 31 (3.3) | 60 (13.9) | 36 (25.7) | 6 (9.2) | 154 (5.1) |
| I am unable to do my usual activities | 21 (1.4) | 3 (0.3) | 14 (3.2) | 6 (4.3) | 34 (52.3) | 78 (2.6) |
| **Pain/Discomfort** | | | | | | |
| I have no pain or discomfort | 698 (47.5) | 198 (21.1) | 43 (10) | 27 (19.3) | 12 (18.5) | 978 (32.1) |
| I have slight pain or discomfort | 579 (39.4) | 507 (54) | 88 (20.4) | 38 (27.1) | 6 (9.2) | 1218 (40.0) |
| I have moderate pain or discomfort | 128 (8.7) | 177 (18.8) | 182 (42.2) | 32 (22.9) | 5 (7.7) | 524 (17.2) |
| I have severe pain or discomfort | 40 (2.7) | 48 (5.1) | 95 (22) | 33 (23.6) | 7 (10.8) | 223 (7.3) |
| I have extreme pain or discomfort | 26 (1.8) | 9 (1) | 23 (5.3) | 10 (7.1) | 35 (53.8) | 103 (3.4) |
| **Anxiety / Depression** | | | | | | |
| I am not anxious or depressed | 752 (51.1) | 248 (26.4) | 52 (12.1) | 32 (22.9) | 11 (16.9) | 1095 (35.9) |
| I am slightly anxious or depressed | 513 (34.9) | 450 (47.9) | 100 (23.2) | 25 (17.9) | 9 (13.8) | 1097 (36.0) |
| I am moderately anxious or depressed | 118 (8) | 175 (18.6) | 163 (37.8) | 15 (10.7) | 3 (4.6) | 474 (15.6) |
| I am severely anxious or depressed | 46 (3.1) | 56 (6) | 88 (20.4) | 53 (37.9) | 9 (13.8) | 252 (8.3) |
| I am extremely anxious or depressed | 42 (2.9) | 10 (1.1) | 28 (6.5) | 15 (10.7) | 33 (50.8) | 128 (4.2) |
|  |  |  |  |  |  |  |
| EQ-VAS score  M (SD); Me (Min,Max) | 82.0 (17.5); 86 (0, 100) | 75.7 (17.4); 80.0 (0, 100) | 68.3 (19.0); 72.0 (0, 100) | 72.4 (19.9); 73.0 (0, 100) | 85.4 (14.0); 88.0 (26, 100) | 77.76 (18.4);  81.0 (0, 100) |
