# Supplementary material for: Prevalence, severity and impacts of breathlessness in Indian adults: An exploratory, nationally representative, cross-sectional online survey
Source: PLOS Glob Public Health. 2024 May 2;4(5):e0002655. doi: 10.1371/journal.pgph.0002655 (PMC11065295; doi:10.1371/journal.pgph.0002655)
Supplement: S3 Table — (DOCX) [file pgph.0002655.s004.docx]

**S3 Table**  Impact of breathlessness measured on the modified Medical Research Council (mMRC ≥1; n=1,351) on respondents’ everyday activities reported in an online survey for India [unweighted data].

|  | mMRC  n (%) | | | | **Total**  **(n=1,575)** |
| --- | --- | --- | --- | --- | --- |
|  | 1  939 (30.8) | 2  431 (14.1) | 3  140 (4.6) | 4  65 (2.1) |  |
| Degree to which breathlessness affects a person’s normal activities of daily life |  | | | | |
| A lot | 118 (12.6) | 144 (33.4) | 54 (38.6) | 42 (64.6) | 358 (22.7) |
| A little | 655 (69.8) | 260 (60.3) | 63 (45) | 15 (23.1) | 993 (63.0) |
| Not at all | 166 (17.7) | 27 (6.3) | 23 (16.4) | 8 (12.3) | 224 (14.2) |
