# Supplementary material for: Prevalence, severity and impacts of breathlessness in Indian adults: An exploratory, nationally representative, cross-sectional online survey
Source: PLOS Glob Public Health. 2024 May 2;4(5):e0002655. doi: 10.1371/journal.pgph.0002655 (PMC11065295; doi:10.1371/journal.pgph.0002655)
Supplement: S4 Table — (DOCX) [file pgph.0002655.s005.docx]

**S4 Table** Mean (SD) and median (IQR) of the World Health Organisation Disability Assessment Schedule 2.0 12-item (WHODAS-12) and its individual domains and breathlessness measured on the modified Medical Research Council (mMRC) breathlessness scale for 3,046 respondents to an online survey in India [unweighted data].

|  | mMRC  n (%) | | | | | **Total**  **(n=3,046)** |
| --- | --- | --- | --- | --- | --- | --- |
|  | **0**  **1,471 (48.3)** | **1**  **939 (30.8)** | **2**  **431 (14.1)** | **3**  **140 (4.6)** | **4**  **65 (2.1)** |  |
| **WHODAS Total score - M(SD); Me (min,max)** | 17.2 (10.1);  17 (0, 48) | 18.9 (8.8);  19 (0, 46) | 26.3 (8.7);  26 (0, 48) | 24.9 (9.6);  25 (0, 44) | 29.5 (10.1);  32 (4, 48) | 19.6 (10.2);  20 (0, 48) |
| 1 – Cognition | 2.9 (1.9);  3 (0, 8) | 3.2 (1.8);  3 (0, 8) | 4.4 (1.8);  4 (0, 8) | 4.2 (2);  4 (0, 8) | 4.7 (1.9);  5 (0, 8) | 3.3 (2);  3 (0, 8) |
| 2 – Mobility | 2.7 (1.9);  2 (0, 8) | 3 (1.7);  3 (0, 8) | 4.3 (1.7);  4 (0, 8) | 3.9 (2);  4 (0, 8) | 4.7 (2.1);  5 (0, 8) | 3.1 (1.9);  3 (0, 8) |
| 3 – Self Care | 2.7 (2.3);  3 (0, 8) | 2.9 (2.1);  3 (0, 8) | 4.4 (2);  4 (0, 8) | 3.9 (2.1);  4 (0, 8) | 5.4 (2.1);  6 (0, 8) | 3.1 (2.3);  3 (0, 8) |
| 4 – Getting along | 3 (2);  3 (0, 8) | 3.2 (1.9);  3 (0, 8) | 4.4 (1.8);  4 (0, 8) | 4.1 (2);  4 (0, 8) | 5.4 (2.2);  6 (0, 8) | 3.3 (2);  3 (0, 8) |
| 5 – Life Activities | 3.1 (2);  3 (0, 8) | 3.4 (1.8);  3 (0, 8) | 4.5 (1.8);  4 (0, 8) | 4.4 (1.7);  5 (0, 8) | 5.2 (2);  6 (0, 8) | 3.5 (2);  4 (0, 8) |
| 6 – Participation | 2.8 (1.9);  3 (0, 8) | 3.2 (1.7);  3 (0, 8) | 4.3 (1.7);  4 (0, 8) | 4.3 (1.9);  5 (0, 8) | 4.2 (2.2);  4 (0, 8) | 3.2 (1.9);  3 (0, 8) |
| Overall, in the past 30 days, how many days were these difficulties present  M(SD); Me (Min,Max) | 11.7 (8.7); 10 (1, 30) | 10.7 (7.3);  8 (1, 30) | 13.9 (7.4); 15 (1, 30) | 12.3 (7.8); 11.5 (1, 30) | 11.9 (6.6);  10 (2, 30) | 11.75 (8.08);  10 (1, 30) |
| In the past 30 days, for how many days were you totally unable to carry out your usual activities or work because of any health condition?  M(SD); Me (Min,Max) | 5.8 (6.3);  4 (0, 29) | 5.2 (4.8);  5 (0, 27) | 7.2 (5.8);  7 (0, 29) | 6.1 (5.6);  5 (0, 26) | 7.3 (5.3);  7 (0, 29) | 5.84 (5.79);  5 (0, 29) |
| In the past 30 days, not counting the days that you were totally unable, for how many days did you cut back or reduce your usual activities or work because of any health condition?  M(SD); Me (Min,Max) | 5.1 (5.7);  3 (0, 29) | 5 (4.7);  5 (0, 29) | 6.2 (5.2);  5 (0, 29) | 5.4 (4.8);  5 (0, 23) | 4.3 (5.3);  3 (0, 29) | 5.23 (5.33);  4 (0, 29) |
