# Supplementary material for: Prevalence, severity and impacts of breathlessness in Indian adults: An exploratory, nationally representative, cross-sectional online survey
Source: PLOS Glob Public Health. 2024 May 2;4(5):e0002655. doi: 10.1371/journal.pgph.0002655 (PMC11065295; doi:10.1371/journal.pgph.0002655)
Supplement: S5 Table — (DOCX) [file pgph.0002655.s006.docx]

**S5 Table**  Population and sample characteristics used for creating sampling weights.

| Characteristics | Population characteristics available | Sample characteristics available |
| --- | --- | --- |
| Age | Age groups   \| 18 \| \| --- \| \| 19 \| \| 20-24 \| \| 25-29 \| \| 30-34 \| \| 35-39 \| \| 40-44 \| \| 45-49 \| \| 50-54 \| \| 55-59 \| \| 60-64 \| \| 65-69 \| \| 70-74 \| \| 75-79 \| \| 80+ \| | Single ages grouped into   \| 18 \| \| --- \| \| 19 \| \| 20-24 \| \| 25-29 \| \| 30-34 \| \| 35-39 \| \| 40-44 \| \| 45-49 \| \| 50-54 \| \| 55-59 \| \| 60-64 \| \| 65-69 \| \| 70-74 \| \| 75-79 \| \| 80+ \| |
| Place of residence | Urban or Rural | Urban or Rural |
| Educational attainment | Literate without education | No formal education |
|  | Below Primary; Primary | Less than 10^th^ grade |
|  | Middle | 10th grade completed |
|  | Matric/Secondary; Higher secondary/Intermediate/Pre-University/Senior secondary; Higher secondary/Intermediate/Pre-University/Senior secondary; Non-technical diploma or certificate not equal to degree; Technical diploma or certificate not equal to degree | 12^th^ grade completed |
|  | Graduate & above | College University completed/ Postgraduate degree completed |
