# Supplementary material for: Prevalence, severity and impacts of breathlessness in Indian adults: An exploratory, nationally representative, cross-sectional online survey
Source: PLOS Glob Public Health. 2024 May 2;4(5):e0002655. doi: 10.1371/journal.pgph.0002655 (PMC11065295; doi:10.1371/journal.pgph.0002655)
Supplement: S6 Table — (DOCX) [file pgph.0002655.s007.docx]

**S6 Table**  India Breathlessness Survey and 2011 Census of India Populations and variables used to create a sample weight.

| Sociodemographic variables | 2011 Census of India Population^*^ (n=513,370,344) | | India Breathlessness Survey  (n=3,046) | | India Breathlessness Survey with Weight |
| --- | --- | --- | --- | --- | --- |
| Age |  |  |  |  |  |
| 18 | 24,230,172 | 4.7 | 48 | 1.6 | 4.9 |
| 19 | 18,280,414 | 3.6 | 69 | 2.3 | 4.5 |
| 20-24 | 92,744,730 | 18.1 | 532 | 17.5 | 24.2 |
| 25-29 | 78,779,525 | 15.3 | 427 | 14.0 | 10.7 |
| 30-34 | 64,265,391 | 12.5 | 345 | 11.3 | 15.5 |
| 35-39 | 57,277,557 | 11.2 | 396 | 13.0 | 15.5 |
| 40-44 | 45,631,240 | 8.9 | 253 | 8.3 | 1.9 |
| 45-49 | 37,240,564 | 7.3 | 330 | 10.8 | 4.4 |
| 50-54 | 28,247,046 | 5.5 | 100 | 3.3 | 8.7 |
| 55-59 | 21,463,974 | 4.2 | 196 | 6.4 | 4.1 |
| 60-64 | 17,674,268 | 3.4 | 112 | 3.7 | 1.3 |
| 65-69 | 11,625,376 | 2.3 | 217 | 7.1 | 1.1 |
| 70-74 | 7,640,218 | 1.5 | 8 | .3 | 2.1 |
| 75-79 | 3,871,311 | 0.8 | 10 | .3 | .9 |
| 80+ | 4,398,558 | 0.9 | 3 | .1 | .2 |
|  |  |  |  |  |  |
| Place of residence |  |  |  |  |  |
| Urban | 209,507,428 | 40.8 | 1034 | 33.9 | 40.8 |
| Rural | 303,862,916 | 59.2 | 2012 | 66.1 | 59.2 |
|  |  |  |  |  |  |
| Educational Attainment |  |  |  |  |  |
| Literate without education | 30,202,161 | 5.9 | 13 | .4 | 5.9 |
| Below Primary; Primary | 157,550,054 | 30.8 | 34 | 1.1 | 30.8 |
| Middle | 89,019,518 | 17.4 | 162 | 5.3 | 17.4 |
| Matric/Secondary; Higher secondary/Intermediate/Pre-University/Senior secondary; Higher secondary/Intermediate/Pre-University/Senior secondary; Non-technical diploma or certificate not equal to degree; Technical diploma or certificate not equal to degree | 166,422,740 | 32.6 | 794 | 26.1 | 32.6 |
| Graduate & above | 68,006,785 | 13.3 | 2,043 | 67.1 | 13.3 |

^*^Of literate population, only
